# Diaqua­bis­(5-methyl-1,2-oxazole-3-car­box­yl­ato-κ^2^ *N*,*O* ^3^)cobalt(II) dihydrate

**DOI:** 10.1107/S1600536811053414

**Published:** 2011-12-21

**Authors:** Yan Wang, Jing Zhao

**Affiliations:** a Ordered Matter Science Research Center, College of Chemistry and Chemical Engineering, Southeast University, Nanjing 210096, People’s Republic of China

## Abstract

In the title compound, [Co(C_5_H_4_NO_3_)_2_(H_2_O)_2_]·2H_2_O, the coordination polyhedron around the six-coordinate Co^II^ ion is formed by two equatorial 5-methyl­isoxazole-3-carboxyl­ate ligands in an *N*,*O* ^3^-bidentate fashion through the isoxazole N atom and a carboxyl­ate O atom, and by two axial water ligands. The asymmetric unit consists of half of the complex and one water mol­ecule (the full comlex being completed by application of inversion). In the crystal, the water mol­ecules participate in the formation of an intricate three-dimensional network of hydrogen bonds involving the coordinated water mol­ecule and the carboxyl­ate groups.

## Related literature

For a related structure, see: Luo *et al.* (2011 ▶).
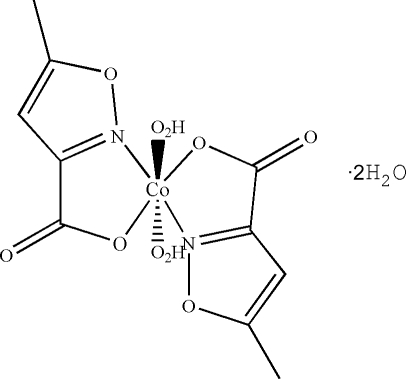


## Experimental

### 

#### Crystal data

[Co(C_5_H_4_NO_3_)_2_(H_2_O)_2_]·2H_2_O
*M* *_r_* = 383.18
Monoclinic, 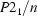

*a* = 5.260 (3) Å
*b* = 18.528 (10) Å
*c* = 8.077 (4) Å
β = 103.707 (6)°
*V* = 764.9 (7) Å^3^
*Z* = 2
Mo *K*α radiation
μ = 1.18 mm^−1^
*T* = 296 K
0.20 × 0.20 × 0.20 mm

#### Data collection

Rigaku SCXmini diffractometer
Absorption correction: multi-scan (*CrystalClear*; Rigaku, 2005 ▶) *T*_min_ = 0.983, *T*_max_ = 0.983
5217 measured reflections
1344 independent reflections
1202 reflections with *I* > 2σ(*I*)
*R*_int_ = 0.034

#### Refinement

*R*[*F* ^2^ > 2σ(*F* ^2^)] = 0.028
*wR*(*F* ^2^) = 0.078
*S* = 1.06
1344 reflections
123 parameters
H atoms treated by a mixture of independent and constrained refinement
Δρ_max_ = 0.28 e Å^−3^
Δρ_min_ = −0.38 e Å^−3^

### 

Data collection: *CrystalClear* (Rigaku, 2005 ▶); cell refinement: *CrystalClear*; data reduction: *CrystalClear*; program(s) used to solve structure: *SHELXS97* (Sheldrick, 2008 ▶); program(s) used to refine structure: *SHELXL97* (Sheldrick, 2008 ▶); molecular graphics: *DIAMOND* (Brandenburg & Putz, 2005 ▶); software used to prepare material for publication: *SHELXL97*.

## Supplementary Material

Crystal structure: contains datablock(s) I, global. DOI: 10.1107/S1600536811053414/vm2142sup1.cif

Structure factors: contains datablock(s) I. DOI: 10.1107/S1600536811053414/vm2142Isup2.hkl

Additional supplementary materials:  crystallographic information; 3D view; checkCIF report

## Figures and Tables

**Table 1:** Hydrogen-bond geometry (Å, °)

*D*—H⋯*A*	*D*—H	H⋯*A*	*D*⋯*A*	*D*—H⋯*A*
O4—H4*A*⋯O1^i^	0.83 (5)	2.07 (5)	2.890 (3)	172 (4)
O4—H4*B*⋯O1^ii^	0.83 (4)	2.03 (4)	2.853 (3)	172 (3)
O3—H3*B*⋯O2^ii^	0.82 (4)	2.07 (4)	2.852 (3)	161 (3)
O3—H3*A*⋯O4	0.76 (3)	1.95 (3)	2.696 (3)	167 (3)

Symmetry codes: (i) 
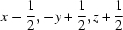
; (ii) 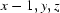.

**Fig. 1.**
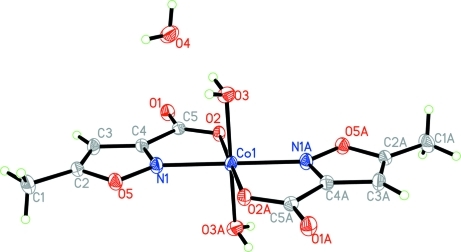
The molecular structure of the title compound with the atom-labelling scheme. Displacement ellipsoids are drawn at the 30% probability level. H atoms are represented as small spheres of arbitrary radii. Symmetry code: (A) -x+1, -y, -z+1.

**Fig. 2.**
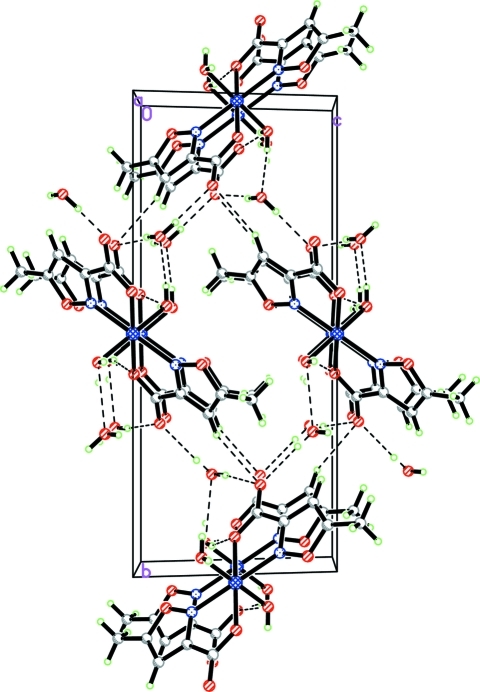
A packing view down the a axis showing the three dimensional network. Intermolecular hydrogen bonds are shown as dashed lines.

## supplementary crystallographic information

### Comment

Isoxazole derivatives are versatile ligands towards transition metal ions both in man-made and natural systems. They are not only used as (bio)catalysts but also for dioxygen transport and electron storage (Luo *et al.*, 2011). As part of our interest in isoxazole derivatives, we report here the crystal structure of a new cobalt complex.

The molecular structure of the title compound is shown in Fig. 1. All non-H atoms, except O3 and O4, are located in the same plane with an r.m.s. deviation of 0.0247 Å.

The coordination polyhedron around the six coordinated central Co^II^ ion is described as a octahedron, formed by two equatorial 5-methylisoxazole-3-carboxylates in an O, N bidentate fashion through the isoxazole nitrogen and the carboxylate oxygen atoms and by two axial water ligands.

The title compound forms a three-dimensional structure *via* intermolecular O—H···O hydrogen bonds interactions (Table 1, Fig. 2).

### Experimental

0.06 g CoCl_2_.6H_2_O (mg) was added to a methanol solution of 0.06 g 5-methyl-3-isoxazolecarboxylic acid and stirred for three h at room temperature. The resulting solution was filtered off and allowed to evaporate at room temperature. Pillar pink crystals of the title compound were obtained within 3 days.

### Refinement

All H atoms attached to C atoms were fixed geometrically and treated as riding with C—H = 0.93 Å (CH), C—H = 0.96 Å (CH_3_) with *U*_iso_(H) = 1.2*U*_eq_(CH) and *U*_iso_(H) = 1.5*U*_eq_(CH_3_). H atoms of water molecules were located in difference Fourier maps and included in the subsequent refinement using restraints (O—H= 0.79 (1) Å with *U*_iso_(H) = 1.5*U*_eq_(O) or *U*_iso_(H) = 2.0 *U*_eq_(O). In the last cycles of refinement, they were treated as riding on their parent O atoms.

### Crystal data

**Table:**

[Co(C_5_H_4_NO_3_)_2_(H_2_O)_2_]·2H_2_O	*F*(000) = 394
*M**_r_* = 383.18	*D*_x_ = 1.664 Mg m^−^^3^
Monoclinic, *P*2_1_/*n*	Mo *K*α radiation, λ = 0.71073 Å
Hall symbol: -P 2yn	Cell parameters from 1344 reflections
*a* = 5.260 (3) Å	θ = 2.2–25.0°
*b* = 18.528 (10) Å	µ = 1.18 mm^−^^1^
*c* = 8.077 (4) Å	*T* = 296 K
β = 103.707 (6)°	Pillar, pink
*V* = 764.9 (7) Å^3^	0.20 × 0.20 × 0.20 mm
*Z* = 2	

### Data collection

**Table:**

Rigaku SCXmini diffractometer	1344 independent reflections
Radiation source: fine-focus sealed tube	1202 reflections with *I* > 2σ(*I*)
graphite	*R*_int_ = 0.034
Detector resolution: 13.6612 pixels mm^-1^	θ_max_ = 25.0°, θ_min_ = 2.2°
CCD_Profile_fitting scans	*h* = −6→6
Absorption correction: multi-scan (*CrystalClear*; Rigaku, 2005)	*k* = −22→22
*T*_min_ = 0.983, *T*_max_ = 0.983	*l* = −9→9
5217 measured reflections	

### Refinement

**Table:**

Refinement on *F*^2^	Primary atom site location: structure-invariant direct methods
Least-squares matrix: full	Secondary atom site location: difference Fourier map
*R*[*F*^2^ > 2σ(*F*^2^)] = 0.028	Hydrogen site location: inferred from neighbouring sites
*wR*(*F*^2^) = 0.078	H atoms treated by a mixture of independent and constrained refinement
*S* = 1.06	*w* = 1/[σ^2^(*F*_o_^2^) + (0.0428*P*)^2^ + 0.2614*P*] where *P* = (*F*_o_^2^ + 2*F*_c_^2^)/3
1344 reflections	(Δ/σ)_max_ < 0.001
123 parameters	Δρ_max_ = 0.28 e Å^−^^3^
0 restraints	Δρ_min_ = −0.38 e Å^−^^3^

### Special details

**Table:**

Geometry. All e.s.d.'s (except the e.s.d. in the dihedral angle between two l.s. planes) are estimated using the full covariance matrix. The cell e.s.d.'s are taken into account individually in the estimation of e.s.d.'s in distances, angles and torsion angles; correlations between e.s.d.'s in cell parameters are only used when they are defined by crystal symmetry. An approximate (isotropic) treatment of cell e.s.d.'s is used for estimating e.s.d.'s involving l.s. planes.
Refinement. Refinement of *F*^2^ against ALL reflections. The weighted *R*-factor *wR* and goodness of fit *S* are based on *F*^2^, conventional *R*-factors *R* are based on *F*, with *F* set to zero for negative *F*^2^. The threshold expression of *F*^2^ > σ(*F*^2^) is used only for calculating *R*-factors(gt) *etc*. and is not relevant to the choice of reflections for refinement. *R*-factors based on *F*^2^ are statistically about twice as large as those based on *F*, and *R*- factors based on ALL data will be even larger.

### Fractional atomic coordinates and isotropic or equivalent isotropic displacement parameters (Å^2^)

**Table:**

	*x*	*y*	*z*	*U*_iso_*/*U*_eq_	
Co1	0.5000	0.0000	0.5000	0.02592 (16)	
O1	0.8288 (3)	0.18878 (7)	0.38174 (19)	0.0361 (4)	
O5	0.0881 (3)	0.06221 (8)	0.16193 (19)	0.0345 (4)	
O2	0.7638 (3)	0.08451 (7)	0.50526 (17)	0.0293 (3)	
N1	0.3171 (3)	0.06356 (9)	0.2896 (2)	0.0312 (4)	
O3	0.3032 (4)	0.05798 (10)	0.6557 (2)	0.0369 (4)	
C5	0.6992 (4)	0.13440 (10)	0.3955 (3)	0.0266 (4)	
C4	0.4387 (4)	0.12311 (10)	0.2705 (3)	0.0270 (4)	
C3	0.2957 (4)	0.16322 (12)	0.1325 (3)	0.0330 (5)	
H3	0.3393	0.2077	0.0937	0.040*	
C1	−0.1485 (5)	0.13125 (15)	−0.0775 (3)	0.0462 (6)	
H1A	−0.1458	0.1782	−0.1275	0.069*	
H1B	−0.3060	0.1259	−0.0382	0.069*	
H1C	−0.1427	0.0949	−0.1612	0.069*	
C2	0.0819 (4)	0.12328 (12)	0.0685 (3)	0.0313 (5)	
O4	0.3191 (4)	0.20264 (10)	0.6234 (3)	0.0511 (5)	
H3A	0.332 (6)	0.0981 (18)	0.655 (4)	0.051 (9)*	
H3B	0.144 (8)	0.0564 (18)	0.623 (5)	0.081 (12)*	
H4B	0.175 (8)	0.2028 (19)	0.555 (5)	0.080 (12)*	
H4A	0.309 (7)	0.232 (2)	0.698 (5)	0.093 (13)*	

### Atomic displacement parameters (Å^2^)

**Table:**

	*U*^11^	*U*^22^	*U*^33^	*U*^12^	*U*^13^	*U*^23^
Co1	0.0265 (2)	0.0213 (2)	0.0275 (3)	−0.00130 (14)	0.00157 (17)	0.00343 (14)
O1	0.0380 (8)	0.0292 (8)	0.0374 (9)	−0.0093 (6)	0.0014 (7)	0.0050 (7)
O5	0.0321 (8)	0.0325 (8)	0.0324 (8)	−0.0032 (6)	−0.0051 (6)	0.0006 (6)
O2	0.0287 (7)	0.0267 (7)	0.0290 (8)	−0.0024 (6)	−0.0002 (6)	0.0046 (6)
N1	0.0300 (9)	0.0284 (9)	0.0301 (10)	−0.0019 (7)	−0.0030 (7)	0.0033 (7)
O3	0.0360 (10)	0.0318 (10)	0.0424 (10)	0.0002 (7)	0.0082 (7)	−0.0041 (7)
C5	0.0281 (10)	0.0244 (10)	0.0269 (11)	−0.0005 (8)	0.0059 (8)	−0.0007 (8)
C4	0.0297 (10)	0.0254 (10)	0.0252 (11)	−0.0006 (8)	0.0052 (8)	0.0007 (8)
C3	0.0374 (11)	0.0294 (11)	0.0305 (11)	0.0014 (9)	0.0048 (9)	0.0085 (9)
C1	0.0409 (13)	0.0579 (16)	0.0329 (13)	0.0074 (11)	−0.0051 (10)	0.0006 (11)
C2	0.0344 (11)	0.0343 (11)	0.0234 (11)	0.0064 (9)	0.0031 (9)	0.0012 (9)
O4	0.0483 (11)	0.0376 (10)	0.0598 (12)	0.0073 (8)	−0.0024 (9)	−0.0118 (9)

### Geometric parameters (Å, °)

**Table:**

Co1—O2^i^	2.0860 (16)	O3—H3B	0.82 (4)
Co1—O2	2.0860 (16)	C5—C4	1.512 (3)
Co1—N1^i^	2.1035 (18)	C4—C3	1.401 (3)
Co1—N1	2.1035 (18)	C3—C2	1.343 (3)
Co1—O3^i^	2.1038 (18)	C3—H3	0.9300
Co1—O3	2.1038 (18)	C1—C2	1.485 (3)
O1—C5	1.236 (2)	C1—H1A	0.9600
O5—C2	1.356 (3)	C1—H1B	0.9600
O5—N1	1.388 (2)	C1—H1C	0.9600
O2—C5	1.270 (2)	O4—H4B	0.83 (4)
N1—C4	1.303 (3)	O4—H4A	0.83 (5)
O3—H3A	0.76 (3)		
			
O2^i^—Co1—O2	180.00 (5)	Co1—O3—H3B	113 (3)
O2^i^—Co1—N1^i^	76.76 (6)	H3A—O3—H3B	103 (3)
O2—Co1—N1^i^	103.24 (6)	O1—C5—O2	126.42 (19)
O2^i^—Co1—N1	103.24 (6)	O1—C5—C4	119.00 (17)
O2—Co1—N1	76.76 (6)	O2—C5—C4	114.57 (16)
N1^i^—Co1—N1	180.0	N1—C4—C3	110.96 (18)
O2^i^—Co1—O3^i^	91.37 (8)	N1—C4—C5	115.51 (17)
O2—Co1—O3^i^	88.63 (8)	C3—C4—C5	133.53 (18)
N1^i^—Co1—O3^i^	90.08 (8)	C2—C3—C4	104.87 (19)
N1—Co1—O3^i^	89.92 (8)	C2—C3—H3	127.6
O2^i^—Co1—O3	88.63 (8)	C4—C3—H3	127.6
O2—Co1—O3	91.37 (8)	C2—C1—H1A	109.5
N1^i^—Co1—O3	89.92 (8)	C2—C1—H1B	109.5
N1—Co1—O3	90.08 (8)	H1A—C1—H1B	109.5
O3^i^—Co1—O3	180.00 (7)	C2—C1—H1C	109.5
C2—O5—N1	107.48 (15)	H1A—C1—H1C	109.5
C5—O2—Co1	117.73 (12)	H1B—C1—H1C	109.5
C4—N1—O5	106.92 (16)	C3—C2—O5	109.77 (18)
C4—N1—Co1	115.26 (13)	C3—C2—C1	134.6 (2)
O5—N1—Co1	137.76 (13)	O5—C2—C1	115.60 (19)
Co1—O3—H3A	112 (2)	H4B—O4—H4A	106 (3)

Symmetry codes: (i) −*x*+1, −*y*, −*z*+1.

### Hydrogen-bond geometry (Å, °)

**Table:**

*D*—H···*A*	*D*—H	H···*A*	*D*···*A*	*D*—H···*A*
O4—H4A···O1^ii^	0.83 (5)	2.07 (5)	2.890 (3)	172 (4)
O4—H4B···O1^iii^	0.83 (4)	2.03 (4)	2.853 (3)	172 (3)
O3—H3B···O2^iii^	0.82 (4)	2.07 (4)	2.852 (3)	161 (3)
O3—H3A···O4	0.76 (3)	1.95 (3)	2.696 (3)	167 (3)

Symmetry codes: (ii) *x*−1/2, −*y*+1/2, *z*+1/2; (iii) *x*−1, *y*, *z*.
